# Effect of different sedatives on the prognosis of patients with mechanical ventilation: a retrospective cohort study based on MIMIC-IV database

**DOI:** 10.3389/fphar.2024.1301451

**Published:** 2024-07-18

**Authors:** Xiaoding Shi, Jiaxing Zhang, Yufei Sun, Meijun Chen, Fei Han

**Affiliations:** ^1^ Department of Anesthesiology, Harbin Medical University Cancer Hospital, Harbin, China; ^2^ College of Bioinformatics Science and Technology, Harbin Medical University, Harbin, China; ^3^ College of 3rd Clinical Medicine, Harbin Medical University, Harbin, China

**Keywords:** mechanical ventilation, ventilator-associated pneumonia, in-hospital mortality, sedative therapy, dexmedetomidine, MIMIC-IV

## Abstract

**Aim:**

To compare the effects of midazolam, propofol, and dexmedetomidine monotherapy and combination therapy on the prognosis of intensive care unit (ICU) patients receiving continuous mechanical ventilation (MV).

**Methods:**

11,491 participants from the Medical Information Mart for Intensive Care (MIMIC)-IV database 2008–2019 was included in this retrospective cohort study. The primary outcome was defined as incidence of ventilator-associated pneumonia (VAP), in-hospital mortality, and duration of MV. Univariate and multivariate logistic regression analyses were utilized to evaluate the association between sedation and the incidence of VAP. Univariate and multivariate Cox analyses were performed to investigate the correlation between sedative therapy and in-hospital mortality. Additionally, univariate and multivariate linear analyses were conducted to explore the relationship between sedation and duration of MV.

**Results:**

Compared to patients not receiving these medications, propofol alone, dexmedetomidine alone, combination of midazolam and dexmedetomidine, combination of propofol and dexmedetomidine, combination of midazolam, propofol and dexmedetomidine were all association with an increased risk of VAP; dexmedetomidine alone, combination of midazolam and dexmedetomidine, combination of propofol and dexmedetomidine, combination of midazolam, propofol and dexmedetomidine may be protective factor for in-hospital mortality, while propofol alone was risk factor. There was a positive correlation between all types of tranquilizers and the duration of MV. Taking dexmedetomidine alone as the reference, all other drug groups were found to be associated with an increased risk of in-hospital mortality. The administration of propofol alone, in combination with midazolam and dexmedetomidine, in combination with propofol and dexmedetomidine, in combination with midazolam, propofol and dexmedetomidine were associated with an increased risk of VAP compared to the use of dexmedetomidine alone.

**Conclusion:**

Dexmedetomidine alone may present as a favorable prognostic option for ICU patients with mechanical ventilation MV.

## Introduction

Mechanical ventilation (MV) is a commonly utilized technique for assisted respiration in patients admitted to intensive care unit (ICU) (Ossai and Wickramasinghe, 2021). However, MV is often associated with various complications and increased mortality (Bezzant and Mortensen, 1994; Goligher et al., 2018). Sedative therapy is often required during clinical treatment to alleviate patient discomfort and stress response, as well as enhance tolerance of mechanical ventilation (MV) (Kawazoe et al., 2017; Heybati et al., 2022). Excessive sedation can result in serious adverse effects for patients, including cardiopulmonary suppression, immunosuppression, increased mortality, and prolonged MV (Sessler and Varney, 2008; Pearson and Patel, 2020). Conversely, inadequate sedation may lead to hypertension and tachycardia (Enomoto et al., 2006). Hence, the selection of a suitable sedation protocol is crucial for optimal patient management.

Currently, midazolam, propofol, and dexmedetomidine are employed sedatives in clinical settings (Jiang and Yan, 2021). A systematic review and meta-analysis of randomized controlled trials has demonstrated that, compared to propofol, dexmedetomidine was associated with a shorter duration of MV, lower incidence of delirium in cardiac surgery patients, and an increased risk of bradycardia across all subsets of ICU patients (Heybati et al., 2022). Zhang et al., also pointed out that dexmedetomidine exhibits potential advantages in reducing the duration of MV and mitigating the risk of delirium (Zhang et al., 2017). The study conducted by Hu et al. demonstrated that in mechanically ventilated ICU patients diagnosed with acute respiratory distress syndrome, the administration of dexmedetomidine for sedation was associated with a lower in-hospital mortality compared to the use of midazolam and propofol (Hu et al., 2021). Ventilator-associated pneumonia (VAP) is a frequently acquired infection in the ICU, which occurs in patients who have undergone continuous MV for more than 48 h, and is strongly associated with high mortality rates, prolonged MV, and increased healthcare costs (Papazian et al., 2020). Dou et al. found that the incidence of VAP among MV patients in the ICU was significantly lower in the combination group of propofol and dexmedetomidine compared to the dexmedetomidine alone group, indicating a different impact of different sedation regimens on VAP occurrence; however, it is important to note that the study had limitations, such as a small sample size and lack of data regarding the effects of propofol alone and other sedatives on VAP (Dou et al., 2020). To our knowledge, there remains a scarcity of research investigating the influence of various sedation regimens on the prognosis of critically ill patients receiving MV (Jakob et al., 2012; Ding et al., 2019; Li et al., 2019).

Herein, this study sought to compare the effects of midazolam, propofol, and dexmedetomidine monotherapy and combination therapy on the incidence of VAP, in-hospital mortality, and duration of MV among ICU patients receiving continuous MV, which provided relevant references for clinicians.

## Methods

### Study populations

In this retrospective cohort study, all data was sourced from the Medical Information Mart for Intensive Care (MIMIC)-IV database 2008–2019. The MIMIC-IV database is a comprehensive and de-identified repository of patient information, encompassing over 70,000 adult ICU admissions from across the United States and made publicly available for research purposes (Peng et al., 2022). The requirement of ethical approval for this was waived by the Institutional Review Board of Harbin Medical University Cancer Hospital, because the data was accessed from MIMIC-IV (a publicly available database). The need for written informed consent was waived by the Institutional Review Board of Harbin Medical University Cancer Hospital due to retrospective nature of the study. All methods were performed in accordance with the relevant guidelines and regulations.

The eligibility criteria for this study were as follows: 1) age of 18 years or older; 2) the use of MV; 3) a minimum ICU stay of 48 h; and 4) complete survival data available. Patients who received MV for less than two consecutive days were excluded. Ultimately, a total of 11,491 participants were deemed eligible for subsequent analysis.

### Exposure

In this study, sedative therapy contains midazolam alone, propofol alone, dexmedetomidine alone, combination of midazolam and propofol, combination of midazolam and dexmedetomidine, combination of propofol and dexmedetomidine, combination of midazolam, propofol and dexmedetomidine.

### Outcomes

The outcome measures comprised the following: incidence of VAP, in-hospital mortality risk, and prolonged duration of MV (>72 h). The median and quartiles of follow-up time was 11.45 (7.13, 18.98) days.

### Data collection

The present study extracted information of patients from MIMIC-IV database, including age, gender, ethnicity, coronary heart disease, sepsis, coronary artery disease (CAD), chronic respiratory failure, acute respiratory failure, effusion, atelectasis, pneumothorax, emphysema, severity scoring system [sequential organ failure assessment (SOFA), charlson comorbidity index (CCI), glasgow coma scale (GCS)], vital signs and laboratory data within 24 h after ICU admission [systolic blood pressure (SBP, mmHg), diastolic blood pressure (DBP, mmHg), respiratory rate (bpm), heart rate (bpm), temperature (°C), white blood cell (WBC, K/uL), platelet (PLT, K/uL), hemoglobin (g/dL), red blood cell distribution width (RDW, %), hematocrit (%), blood urea nitrogen (BUN, mg/dL), creatinine (mg/dL), glucose (mg/dL), sodium (mEq/L), chloride (mEq/L), bicarbonate (mEq/L)], intervention means [vasopressors, renal replacement therapy (RRT), oral care, opioids]. Only data from the initial ICU admission was used for patients who were admitted to the ICU on multiple occasions.

### Statistical analysis

Multiple imputation method was employed to handle missing values (Zhang et al., 2016), and sensitivity analyses were conducted on the data both pre- and post-treatment (Supplemental Table S1). The categorical data were presented as the number of cases and the constituent ratio [n (%)]. Mean ± standard deviation (Mean ± SD) is utilized to describe the normal distribution of the measured data, while median and quartiles [M (Q1, Q3)] is employed to describe the non-normal distribution.

For this study, we assessed the effects of midazolam alone, propofol alone, dexmedetomidine alone, combination of midazolam and propofol, combination of midazolam and dexmedetomidine, combination of propofol and dexmedetomidine, combination of midazolam, propofol and dexmedetomidine on three outcomes (incidence of VAP, in-hospital mortality risk, and duration of MV), respectively. Initially, we employed the Least Absolute Shrinkage and Selection Operator (LASSO) analyses to identify potential confounders associated with the risk of VAP, in-hospital mortality, and duration of MV, respectively. Secondly, univariate and multivariate logistic regression analyses were utilized to evaluate the association between sedation and the incidence of VAP, as well as the duration of MV > 72 h, with calculation of odds ratio (OR) and 95% confidence interval (CI). Univariate and multivariate Cox analyses were performed to investigate the correlation between sedative therapy and in-hospital mortality, with calculation of hazard ratio (HR) and 95% CI. *P* < 0.05 was considered statistically significant. Additionally, subgroup analysis was performed based on the COPD (Yes/No), acute respiratory failure (Yes/No), and sepsis (Yes/No).

## Results

### Characteristics of participants

**FIGURE 1:**
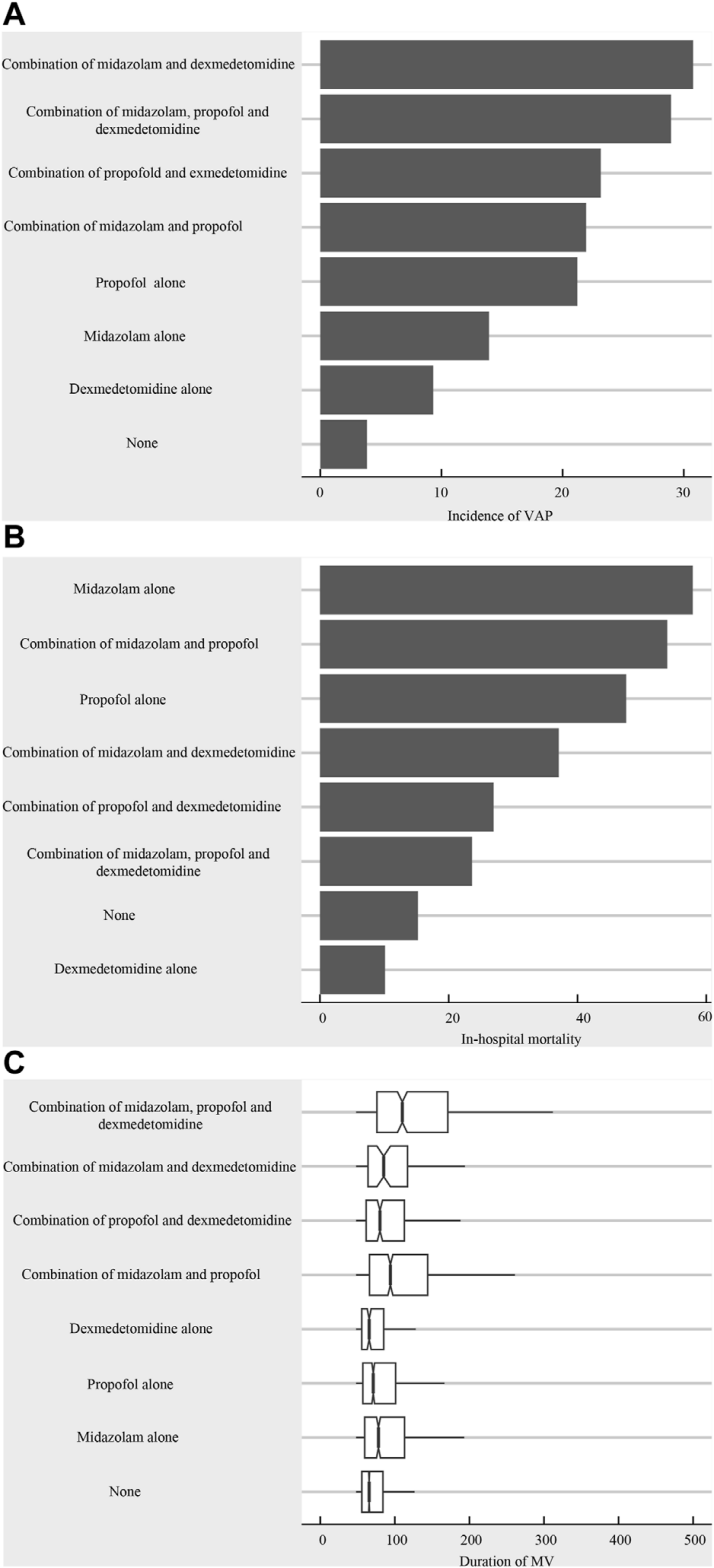
**(A)** The incidence of VAP across different sedative treatment cohorts; **(B)** In-hospital mortality risk of different sedative treatment cohorts; **(C)** The duration of MV in the different sedative treatment cohorts.

The characteristics of the subjects under study are summarized in Table 1 (n = 11,491). 3,047 patients received midazolam, while propofol was administered to 3,969 patients, and dexmedetomidine was used in 1,461 cases. The overall mean age was 65.05 ± 15.89 years, and in-hospital mortality was 21.11% (n = 2,426). The participants’ characteristics in different sedation also shown in Table 1. In addition, we also analyzed the development of VAP and in-hospital mortality among different sedative therapy cohorts. As shown in Table 2, 11.20% patients who received midazolam alone developed VAP, and the in-hospital mortality was 15.70%. Similarly, the incidence of VAP in patients treated with propofol alone, dexmedetomidine alone, midazolam combined with dexmedetomidine, midazolam combined with propofol, propofol combined with dexmedetomidine, and midazolam combined with propofol and dexmedetomidine was 23.37%, 2.29%, 1.76%, 21.96%, 8.82% and 10.67%, respectively. The incidence of VAP, in-hospital mortality risk, and duration of MV exhibited variation across the various sedative treatment cohorts (Figures 1A–C).

**TABLE 1:** Characteristics of participants and comparison of different sedatives.

Variables	Total (n = 11,491)	Sedated with midazolam (n = 3,047)	Sedated with propofol (n = 3,969)	Sedated with dexmedetomidine (n = 1,461)
Characteristics				
Age, year, Mean ± SD	65.05 ± 15.89	60.65 ± 16.50	59.74 ± 16.65	58.65 ± 16.43
Gender, female, n (%)	5,073 (44.15)	1,268 (41.61)	1,609 (40.54)	516 (35.32)
Race, n (%)				
White	7,714 (67.13)	1900 (62.36)	2,339 (58.93)	883 (60.44)
Black	968 (8.42)	299 (9.81)	402 (10.13)	130 (8.90)
Asian	291 (2.53)	85 (2.79)	94 (2.37)	32 (2.19)
Other	2,518 (21.91)	763 (25.04)	1,134 (28.57)	416 (28.47)
Coronary heart disease, yes, n (%)	3,402 (29.61)	726 (23.83)	879 (22.15)	353 (24.16)
Sepsis, yes, n (%)	4,944 (43.02)	2013 (66.06)	2,619 (65.99)	935 (64.00)
CAD, yes, n (%)	3,402 (29.61)	726 (23.83)	879 (22.15)	353 (24.16)
COPD, yes, n (%)	1,113 (9.69)	253 (8.30)	394 (9.93)	199 (13.62)
Chronic respiratory failure, yes, n (%)	51 (0.44)	11 (0.36)	4 (0.10)	0 (0.00)
Acute respiratory failure, yes, n (%)	2,576 (22.42)	1,360 (44.63)	1,185 (29.86)	240 (16.43)
Effusion, yes, n (%)	943 (8.21)	320 (10.50)	314 (7.91)	77 (5.27)
Atelectasis, yes, n (%)	379 (3.30)	57 (1.87)	143 (3.60)	87 (5.95)
Pneumothorax, yes, n (%)	534 (4.65)	180 (5.91)	252 (6.35)	85 (5.82)
Emphysema, yes, n (%)	373 (3.25)	97 (3.18)	122 (3.07)	48 (3.29)
Scoring systems				
SOFA, score, M (Q_1_, Q_3_)	7.00 (4.00, 12.00)	11.00 (8.00, 15.00)	11.00 (8.00, 14.00)	10.00 (7.00, 13.00)
GCS, score, M (Q_1_, Q_3_)	12.00 (7.00, 14.00)	9.00 (3.00, 13.00)	9.00 (4.00, 13.00)	10.00 (6.00, 13.00)
CCI, score, M (Q_1_, Q_3_)	3.00 (1.00, 5.00)	3.00 (1.00, 5.00)	3.00 (1.00, 4.00)	2.00 (1.00, 4.00)
Vital signs				
Heart rate, bpm, Mean ± SD	90.85 ± 20.68	94.60 ± 22.05	91.78 ± 21.41	92.99 ± 20.75
SBP, mmHg, Mean ± SD	119.13 ± 24.52	117.35 ± 25.39	119.79 ± 25.39	118.66 ± 23.49
DBP, mmHg, Mean ± SD	65.06 ± 17.17	65.82 ± 17.70	66.81 ± 17.72	67.36 ± 17.99
Respiratory rate, times/min, Mean ± SD	20.65 ± 6.05	21.71 ± 6.24	21.04 ± 6.13	21.16 ± 6.24
Temperature, °C, Mean ± SD	36.72 ± 0.95	36.71 ± 1.16	36.73 ± 1.10	36.86 ± 0.99
Laboratory value				
WBC, K/uL, M (Q_1_, Q_3_)	11.90 (8.50, 16.60)	12.30 (8.20, 18.00)	12.00 (8.30, 17.20)	12.30 (8.70, 17.10)
PLT, K/uL, M (Q_1_, Q_3_)	187.00 (131.00, 257.00)	189.00 (127.00, 265.00)	181.00 (125.00, 252.00)	175.00 (127.00, 243.00)
Hemoglobin, g/dL, Mean ± SD	10.52 ± 2.18	10.69 ± 2.34	10.71 ± 2.34	10.68 ± 2.32
RDW, %, Mean ± SD	15.38 ± 2.39	15.43 ± 2.31	15.32 ± 2.38	15.17 ± 2.36
Hematocrit, %, Mean ± SD	32.10 ± 6.50	32.65 ± 7.10	32.62 ± 7.01	32.71 ± 7.03
BUN, mg/dL, M (Q_1_, Q_3_)	21.00 (14.00, 36.00)	24.00 (15.00, 38.00)	21.00 (14.00, 34.00)	19.00 (13.00, 31.00)
Creatinine, mg/dL, M (Q_1_, Q_3_)	1.10 (0.70, 1.70)	1.20 (0.80, 1.90)	1.10 (0.80, 1.70)	1.00 (0.70, 1.60)
Glucose, mg/dL, M (Q_1_, Q_3_)	135.00 (110.00, 173.00)	142.00 (111.00, 188.00)	140.00 (112.00, 182.00)	137.00 (112.00, 176.00)
Sodium, mEq/L, Mean ± SD	138.26 ± 5.33	138.40 ± 5.71	138.58 ± 5.54	138.39 ± 5.45
Chloride, mEq/L, Mean ± SD	103.91 ± 6.76	104.39 ± 7.07	104.29 ± 6.91	103.83 ± 6.65
Bicarbonate, mEq/L, Mean ± SD	22.94 ± 5.06	22.04 ± 5.59	22.13 ± 5.34	22.32 ± 5.08
Interventions				
Vasopressors, yes, n (%)	5,552 (48.32)	2,312 (75.88)	2,827 (71.23)	994 (68.04)
Renal replacement therapy, yes, n (%)	1714 (14.92)	721 (23.66)	869 (21.89)	271 (18.55)
Oral care, yes, n (%)	11,185 (97.34)	3,043 (99.87)	3,963 (99.85)	1,441 (98.63)
Opioids, yes, n (%)	7,545 (65.66)	2,973 (97.57)	3,640 (91.71)	1,301 (89.05)
Outcomes				
VAP, yes, n (%)	1,134 (9.87)	517 (16.97)	735 (18.52)	267 (18.28)
Duration of MV, hours, M (Q_1_, Q_3_)	70.50 (57.00, 99.00)	91.00 (64.00, 138.00)	84.00 (62.00, 128.00)	85.00 (63.00, 129.00)
In-hospital mortality, yes, n (%)	2,426 (21.11)	992 (32.56)	1,189 (29.96)	267 (18.28)

CAD, coronary artery disease; COPD, chronic obstructive pulmonary disease; SOFA, sequential organ failure assessment; CCI, charlson comorbidity index; GCS, glasgow coma scale; SBP, systolic blood pressure; DBP, diastolic blood pressure; WBC, white blood cell; PLT, platelet; RDW, red blood cell distribution width; BUN, blood urea nitrogen; VAP, ventilator-associated pneumonia; MV, mechanical ventilation.

**TABLE 2:** The development of VAP and in-hospital mortality among different sedative therapy cohorts.

Variables, n (%)	Total (n = 11,491)	VAP		In-hospital mortality	
		No (n = 10,357)	Yes (n = 1,134)	No (n = 9,065)	Yes (n = 2,426)
None	6,093 (53.02)	5,867 (56.65)	226 (19.93)	5,288 (58.33)	805 (33.18)
Midazolam alone (yes)	1,039 (9.04)	912 (8.81)	127 (11.20)	658 (7.26)	381 (15.70)
Propofol alone (yes)	1,514 (13.18)	1,249 (12.06)	265 (23.37)	1,026 (11.32)	488 (20.12)
Dexmedetomidine alone (yes)	305 (2.65)	279 (2.69)	26 (2.29)	277 (3.06)	28 (1.15)
Combination of midazolam and dexmedetomidine (yes)	1,384 (12.04)	1,135 (10.96)	249 (21.96)	899 (9.92)	485 (19.99)
Combination of midazolam and propofol (yes)	85 (0.74)	65 (0.63)	20 (1.76)	62 (0.68)	23 (0.95)
Combination of propofol and dexmedetomidine (yes)	532 (4.63)	432 (4.17)	100 (8.82)	419 (4.62)	113 (4.66)
Combination of midazolam, propofol and dexmedetomidine (yes)	539 (4.69)	418 (4.04)	121 (10.67)	436 (4.81)	103 (4.25)

VAP, ventilator-associated pneumonia.

### Screening of confounding factors

Through LASSO analyses, we identified potential confounding factors associated with three outcomes (Supplementary Figures S1A–S1C). When VAP is the outcome, confounding factors include age, gender, ethnicity, coronary heart disease, sepsis, CAD, chronic respiratory failure, acute respiratory failure, COPD, pneumothorax, emphysema, SOFA, CCI, GCS, SBP, DBP, heart rate, respiratory rate, temperature, WBC, PLT, hemoglobin, RDW, hematocrit, BUN, creatinine, sodium, bicarbonate, vasopressors, oral care, and opioids. Similarly, when in-hospital mortality is the outcome, age, gender, ethnicity, sepsis, CAD, acute respiratory failure, chronic respiratory failure, COPD, effusion, atelectasis, SOFA, CCI, GCS, SBP, DBP, respiratory rate, temperature, WBC, PLT, RDW, hematocrit, BUN, creatinine, glucose, sodium, chloride, bicarbonate, vasopressors, RRT, oral care, and opioids are considered as confounding factors. Taking duration of MV as the outcome variable, age, gender, sepsis, CAD, chronic respiratory failure, acute respiratory failure, COPD, atelectasis, pneumothorax, SOFA, CCI, GCS, heart rate, SBP, respiratory rate, PLT, hematocrit, creatinine, chloride, vasopressors, RRT, oral care, and opioids are considered as confounding factors.

### Effects of different sedative therapy cohorts on three outcomes: incidence of VAP, in-hospital mortality, and duration of MV

In logistic regression analyses (Table 3), we found that propofol alone (Model 2: OR = 1.77, 95% CI: 1.51–2.08; *P* < 0.001), dexmedetomidine alone (Model 2: OR = 1.41, 95% CI: 1.19–1.68; *P* < 0.001), combination of midazolam and dexmedetomidine (Model 2: OR = 1.41, 95% CI: 1.13–1.77; *P* = 0.002), combination of propofol and dexmedetomidine (Model 2: OR = 1.42, 95% CI: 1.18–1.71; *P* < 0.001), combination of midazolam, propofol and dexmedetomidine (Model 2: OR = 1.35, 95% CI: 1.06–1.71; *P* = 0.013) were all associated with an increased risk of VAP.

**TABLE 3:** Impact of various sedatives administered alone or in combination on the incidence of VAP, in-hospital mortality, and duration of MV.

Variables	VAP^I^		In-hospital mortality ^Ⅱ^		Duration of MV ^Ⅲ^	
	OR (95% CI)	*P*	HR (95% CI)	*P*	OR (95% CI)	*P*
Model 1						
Midazolam alone (yes)	2.59 (2.29–2.94)	<0.001	1.55 (1.43–1.68)	<0.001	2.86 (2.62–3.11)	<0.001
Propofol alone (yes)	4.06 (3.57–4.61)	<0.001	1.30 (1.20–1.41)	<0.001	2.30 (2.12–2.49)	<0.001
Dexmedetomidine alone (yes)	2.36 (2.03–2.75)	<0.001	0.63 (0.56–0.72)	<0.001	1.91 (1.70–2.13)	<0.001
Combination of midazolam and propofol (yes)	2.75 (2.40–3.14)	<0.001	1.21 (1.11–1.33)	<0.001	3.41 (3.06–3.80)	<0.001
Combination of midazolam and dexmedetomidine (yes)	2.90 (2.38–3.54)	<0.001	0.68 (0.57–0.82)	<0.001	3.84 (3.18–4.64)	<0.001
Combination of propofol and dexmedetomidine (yes)	2.71 (2.30–3.19)	<0.001	0.68 (0.59–0.78)	<0.001	2.61 (2.28–2.98)	<0.001
Combination of midazolam, propofol and dexmedetomidine (yes)	2.84 (2.30–3.51)	<0.001	0.63 (0.52–0.77)	<0.001	4.29 (3.49–5.29)	<0.001
Model 2						
Midazolam alone (yes)	1.12 (0.96–1.32)	0.162	1.11 (1.01–1.22)	0.050	1.74 (1.56–1.93)	<0.001
Propofol alone (yes)	1.77 (1.51–2.08)	<0.001	1.11 (1.01–1.22)	0.034	1.27 (1.15–1.40)	<0.001
Dexmedetomidine alone (yes)	1.41 (1.19–1.68)	<0.001	0.61 (0.53–0.69)	<0.001	1.31 (1.16–1.49)	<0.001
Combination of midazolam and propofol (yes)	1.16 (0.99–1.36)	0.071	1.00 (0.90–1.11)	0.937	2.02 (1.79–2.28)	<0.001
Combination of midazolam and dexmedetomidine (yes)	1.41 (1.13–1.77)	0.002	0.64 (0.53–0.77)	<0.001	2.30 (1.89–2.81)	<0.001
Combination of propofol and dexmedetomidine (yes)	1.42 (1.18–1.71)	<0.001	0.64 (0.55–0.74)	<0.001	1.63 (1.41–1.88)	<0.001
Combination of midazolam, propofol and dexmedetomidine (yes)	1.35 (1.06–1.71)	0.013	0.61 (0.50–0.75)	<0.001	2.58 (2.07–3.21)	<0.001

VAP, ventilator-associated pneumonia; MV, mechanical ventilation; OR, odds ratio; HR, hazard ratio; CI, confidence interval. Model 1: no adjustment for confounding factors. Model 2: Ⅰ: adjusted age, gender, ethnicity, coronary heart disease, sepsis, coronary artery disease (CAD), chronic respiratory failure, acute respiratory failure, chronic obstructive pulmonary disease (COPD), pneumothorax, emphysema, sequential organ failure assessment (SOFA), charlson comorbidity index (CCI), glasgow coma scale (GCS), systolic blood pressure (SBP), diastolic blood pressure (DBP), heart rate, respiratory rate, temperature, white blood cell (WBC), platelet (PLT), hemoglobin, red blood cell distribution width (RDW), hematocrit, blood urea nitrogen (BUN), creatinine, sodium, bicarbonate, vasopressors, oral care, and opioids. II: adjusted age, gender, ethnicity, sepsis, acute respiratory failure, CAD, COPD, effusion, atelectasis, SOFA, CCI, GCS, SBP, DBP, respiratory rate, temperature, WBC, PLT, RDW, hematocrit, BUN, creatinine, glucose, sodium, chloride, bicarbonate, vasopressors, renal replacement therapy (RRT), oral care, and opioids. Ⅲ: adjusted age, gender, sepsis, CAD, chronic respiratory failure, acute respiratory failure, COPD, atelectasis, pneumothorax, SOFA, CCI, GCS, heart rate, SBP, respiratory rate, PLT, hematocrit, creatinine, chloride, vasopressors, RRT, oral care, and opioids.

Through Cox analyses (Table 3), dexmedetomidine alone (Model 2: HR = 0.61, 95% CI: 0.53–0.69; *P* < 0.001), combination of midazolam and dexmedetomidine (Model 2: HR = 0.64, 95% CI: 0.53–0.77; *P* < 0.001), combination of propofol and dexmedetomidine (Model 2: HR = 0.64, 95% CI: 0.55–0.74; *P* < 0.001), combination of midazolam, propofol and dexmedetomidine (Model 2: HR = 0.61, 95% CI: 0.50–0.75; *P* < 0.001) may be protective factor for in-hospital mortality, while propofol alone (Model 2: HR = 1.11, 95% CI: 1.01–1.22; *P* = 0.034) was risk factor. Likewise, there was a positive correlation between all types of tranquilizers and the duration of MV > 72 h by logistic regression analyses (*P* < 0.05) (Table 3).

Additionally, as shown in Table 4, the administration of propofol alone, in combination with midazolam and dexmedetomidine, in combination with propofol and dexmedetomidine, and in combination with midazolam, propofol and dexmedetomidine were all found to be associated with an increased risk of VAP and increased in-hospital mortality compared to the use of dexmedetomidine alone. Compared with dexmedetomidine alone administration, other sedative therapy cohorts were related to the increased risk of prolonged duration of MV.

**TABLE 4:** Association of various sedatives administered alone or combination and incidence of VAP, in-hospital mortality, and duration of MV (exclude participants who were not sedated).

Variables	VAP^I^		In-hospital mortality^Ⅱ^		Duration of MV^Ⅲ^	
	OR (95% CI)	*P*	HR (95% CI)	*P*	OR (95% CI)	*P*
Model 1						
Dexmedetomidine	Ref		Ref		Ref	
Midazolam	1.49 (0.96–2.33)	0.075	4.18 (2.85–6.13)	<0.001	2.28 (1.75–2.97)	<0.001
Propofol	2.28 (1.49–3.48)	<0.001	3.18 (2.17–4.65)	<0.001	1.65 (1.28–2.12)	<0.001
Combination of midazolam and propofol	2.35 (1.54–3.60)	<0.001	3.45 (2.36–5.05)	<0.001	3.95 (3.04–5.12)	<0.001
Combination of midazolam and dexmedetomidine	3.30 (1.74–6.28)	<0.001	2.59 (1.49–4.50)	<0.001	3.09 (1.87–5.09)	<0.001
Combination of propofol and dexmedetomidine	2.48 (1.57–3.92)	<0.001	1.91 (1.26–2.89)	0.002	2.46 (1.84–3.28)	<0.001
Combination of midazolam, propofol and dexmedetomidine	3.11 (1.98–4.87)	<0.001	1.63 (1.07–2.47)	0.022	6.46 (4.74–8.82)	<0.001
Model 2						
Dexmedetomidine	Ref		Ref		Ref	
Midazolam	1.17 (0.71–1.92)	0.537	3.16 (2.11–4.71)	<0.001	1.57 (1.17–2.12)	0.003
Propofol	1.63 (1.03–2.58)	0.036	2.88 (1.96–4.25)	<0.001	1.15 (0.87–1.51)	0.333
Combination of midazolam and propofol	1.45 (0.90–2.33)	0.127	3.04 (2.04–4.52)	<0.001	2.29 (1.71–3.07)	<0.001
Combination of midazolam and dexmedetomidine	2.29 (1.14–4.61)	0.020	2.11 (1.20–3.71)	0.009	1.93 (1.13–3.28)	0.016
Combination of propofol and dexmedetomidine	1.69 (1.03–2.78)	0.038	1.81 (1.18–2.76)	0.006	1.61 (1.18–2.21)	0.003
Combination of midazolam, propofol and dexmedetomidine	1.89 (1.14–3.11)	0.013	1.62 (1.05–2.50)	0.028	3.77 (2.68–5.30)	<0.001

VAP, ventilator-associated pneumonia; MV, mechanical ventilation; OR, odds ratio; HR, hazard ratio; CI, confidence interval. Model 1: no adjustment for confounding factors. Model 2: Ⅰ: adjusted age, gender, ethnicity, coronary heart disease, sepsis, coronary artery disease (CAD), chronic respiratory failure, acute respiratory failure, chronic obstructive pulmonary disease (COPD), pneumothorax, emphysema, sequential organ failure assessment (SOFA), charlson comorbidity index (CCI), glasgow coma scale (GCS), systolic blood pressure (SBP), diastolic blood pressure (DBP), heart rate, respiratory rate, temperature, white blood cell (WBC), platelet (PLT), hemoglobin, red blood cell distribution width (RDW), hematocrit, blood urea nitrogen (BUN), creatinine, sodium, bicarbonate, vasopressors, oral care, and opioids. Ⅱ: adjusted age, gender, ethnicity, sepsis, acute respiratory failure, CAD, COPD, effusion, atelectasis, SOFA, CCI, GCS, SBP, DBP, respiratory rate, temperature, WBC, PLT, RDW, hematocrit, BUN, creatinine, glucose, sodium, chloride, bicarbonate, vasopressors, renal replacement therapy (RRT), oral care, and opioids. Ⅲ: adjusted age, gender, sepsis, CAD, chronic respiratory failure, acute respiratory failure, COPD, atelectasis, pneumothorax, SOFA, CCI, GCS, heart rate, SBP, respiratory rate, PLT, hematocrit, creatinine, chloride, vasopressors, RRT, oral care, and opioids.

### Subgroup analysis

To assess the effects of midazolam, propofol, and dexmedetomidine monotherapy and combination therapy on the prognosis of ICU patients receiving continuous MV, stratified analyses were conducted based on COPD (Yes/No), acute respiratory failure (Yes/No), and sepsis (Yes/No). In the Table 5, for patients without COPD, acute respiratory failure and sepsis, the administration of sedatives may potentially increase the risk of VAP and duration of MV > 72 h, decrease in-hospital mortality. For patients with COPD, dexmedetomidine alone was related to higher risk of VAP (OR = 1.76, 95% CI: 1.04–2.97; *P* = 0.035), lower risk of in-hospital mortality (HR = 0.51, 95% CI: 0.36–0.73; *P* < 0.001) compared to non-dexmedetomidine treatment. For patients with acute respiratory failure, we found the association of propofol alone and risk of VAP (OR = 1.44, 95% CI: 1.11–1.85; *P* = 0.005) compared to non-propofol treatment.

**TABLE 5:** Subgroup analysis about the impact of various sedatives administered alone or in combination on the incidence of VAP, in-hospital mortality, and duration of MV.

Variables	VAP^I^		In-hospital mortality^Ⅱ^		Duration of MV^Ⅲ^	
	OR (95% CI)	*P*	HR (95% CI)	*P*	OR (95% CI)	*P*
COPD: No (n = 10,378)						
Midazolam alone (yes)	1.13 (0.95–1.33)	0.162	1.11 (1.00–1.23)	0.057	1.75 (1.56–1.95)	<0.001
Propofol alone (yes)	1.79 (1.51–2.11)	<0.001	1.10 (0.99–1.22)	0.068	1.27 (1.15–1.41)	<0.001
Dexmedetomidine alone (yes)	1.38 (1.15–1.65)	<0.001	0.62 (0.53–0.71)	<0.001	1.37 (1.20–1.56)	<0.001
Combination of midazolam and propofol (yes)	1.18 (1.00–1.40)	0.051	0.99 (0.89–1.11)	0.874	2.04 (1.80–2.32)	<0.001
Combination of midazolam and dexmedetomidine (yes)	1.38 (1.09–1.75)	0.007	0.64 (0.53–0.78)	<0.001	2.34 (1.89–2.89)	<0.001
Combination of propofol and dexmedetomidine (yes)	1.39 (1.14–1.70)	0.001	0.64 (0.54–0.75)	<0.001	1.69 (1.45–1.98)	<0.001
Combination of midazolam, propofol and dexmedetomidine (yes)	1.32 (1.03–1.70)	0.031	0.61 (0.49–0.76)	<0.001	2.59 (2.05–3.28)	<0.001
COPD: yes (n = 1,113)						
Midazolam alone (yes)	1.02 (0.58–1.78)	0.943	1.20 (0.86–1.66)	0.284	1.63 (1.15–2.32)	0.007
Propofol alone (yes)	1.52 (0.83–2.77)	0.173	1.13 (0.81–1.58)	0.469	1.21 (0.86–1.69)	0.267
Dexmedetomidine alone (yes)	1.76 (1.04–2.97)	0.035	0.51 (0.36–0.73)	<.001	1.04 (0.73–1.48)	0.821
Combination of midazolam and propofol (yes)	0.92 (0.52–1.66)	0.792	1.04 (0.73–1.48)	0.845	1.77 (1.17–2.68)	0.006
Combination of midazolam and dexmedetomidine (yes)	1.53 (0.76–3.06)	0.231	0.57 (0.34–0.96)	0.034	2.17 (1.21–3.91)	0.010
Combination of propofol and dexmedetomidine (yes)	1.60 (0.92–2.76)	0.095	0.58 (0.40–0.83)	0.003	1.31 (0.87–1.96)	0.195
Combination of midazolam, propofol and dexmedetomidine (yes)	1.44 (0.69–2.98)	0.331	0.59 (0.34–1.02)	0.060	2.55 (1.31–4.95)	0.006
Acute respiratory failure: no (n = 8,915)						
Midazolam alone (yes)	1.20 (0.99–1.46)	0.062	1.15 (1.02–1.30)	0.028	1.63 (1.43–1.85)	<0.001
Propofol alone (yes)	1.95 (1.58–2.41)	<0.001	1.11 (0.98–1.27)	0.104	1.34 (1.19–1.51)	<0.001
Dexmedetomidine alone (yes)	1.44 (1.19–1.75)	<0.001	0.62 (0.53–0.71)	<0.001	1.29 (1.13–1.48)	<0.001
Combination of midazolam and propofol (yes)	1.18 (0.96–1.45)	0.112	0.95 (0.83–1.09)	0.456	1.96 (1.69–2.28)	<0.001
Combination of midazolam and dexmedetomidine (yes)	1.43 (1.10–1.86)	0.008	0.69 (0.56–0.85)	<0.001	2.42 (1.91–3.05)	<0.001
Combination of propofol and dexmedetomidine (yes)	1.49 (1.21–1.85)	<0.001	0.65 (0.56–0.77)	<0.001	1.67 (1.42–1.97)	<0.001
Combination of midazolam, propofol and dexmedetomidine (yes)	1.39 (1.06–1.84)	0.019	0.65 (0.51–0.81)	<0.001	2.70 (2.10–3.49)	<0.001
Acute respiratory failure: yes (n = 2,576)						
Midazolam alone (yes)	1.05 (0.79–1.41)	0.733	1.00 (0.85–1.19)	0.958	1.96 (1.60–2.41)	<0.001
Propofol alone (yes)	1.44 (1.11–1.85)	0.005	0.98 (0.84–1.13)	0.745	1.16 (0.97–1.39)	0.099
Dexmedetomidine alone (yes)	1.18 (0.82–1.70)	0.383	0.43 (0.30–0.60)	<0.001	1.53 (1.13–2.07)	0.006
Combination of midazolam and propofol (yes)	1.12 (0.86–1.47)	0.386	0.99 (0.84–1.17)	0.937	2.10 (1.70–2.58)	<0.001
Combination of midazolam and dexmedetomidine (yes)	1.27 (0.83–1.96)	0.268	0.39 (0.26–0.60)	<0.001	2.09 (1.41–3.08)	<0.001
Combination of propofol and dexmedetomidine (yes)	1.05 (0.70–1.59)	0.804	0.41 (0.28–0.60)	<0.001	1.61 (1.14–2.27)	0.006
Combination of midazolam, propofol and dexmedetomidine (yes)	1.12 (0.70–1.81)	0.634	0.39 (0.25–0.62)	<0.001	2.35 (1.50–3.69)	<0.001
Sepsis: No (n = 6,547)						
Midazolam alone (yes)	1.47 (0.91–2.38)	0.119	1.22 (1.03–1.43)	0.019	1.53 (1.30–1.79)	<0.001
Propofol alone (yes)	2.05 (1.31–3.21)	0.002	1.32 (1.14–1.53)	<0.001	1.10 (0.95–1.27)	0.209
Dexmedetomidine alone (yes)	2.13 (1.31–3.47)	0.002	0.35 (0.26–0.47)	<0.001	1.23 (1.02–1.49)	0.034
Combination of midazolam and propofol (yes)	1.33 (0.79–2.25)	0.277	1.14 (0.95–1.36)	0.155	1.73 (1.43–2.09)	<0.001
Combination of midazolam and dexmedetomidine (yes)	2.70 (1.42–5.15)	0.002	0.33 (0.21–0.53)	<0.001	1.87 (1.36–2.56)	<0.001
Combination of propofol and dexmedetomidine (yes)	2.77 (1.63–4.69)	<0.001	0.32 (0.22–0.46)	<0.001	1.40 (1.10–1.78)	0.006
Combination of midazolam, propofol and dexmedetomidine (yes)	2.88 (1.47–5.62)	0.002	0.26 (0.15–0.47)	<0.001	1.84 (1.30–2.58)	<0.001
Sepsis: Yes (n = 4,944)						
Midazolam alone (yes)	1.08 (0.91–1.28)	0.362	1.07 (0.94–1.22)	0.302	1.86 (1.60–2.16)	<0.001
Propofol alone (yes)	1.70 (1.43–2.02)	<0.001	0.98 (0.87–1.11)	0.775	1.39 (1.21–1.60)	<0.001
Dexmedetomidine alone (yes)	1.32 (1.11–1.58)	0.002	0.74 (0.63–0.86)	<0.001	1.35 (1.14–1.59)	<0.001
Combination of midazolam and propofol (yes)	1.14 (0.97–1.35)	0.116	0.96 (0.84–1.10)	0.572	2.17 (1.84–2.55)	<0.001
Combination of midazolam and dexmedetomidine (yes)	1.31 (1.03–1.65)	0.025	0.77 (0.63–0.95)	0.015	2.53 (1.93–3.31)	<0.001
Combination of propofol and dexmedetomidine (yes)	1.29 (1.06–1.57)	0.010	0.78 (0.66–0.91)	0.003	1.72 (1.42–2.09)	<0.001
Combination of midazolam, propofol and dexmedetomidine (yes)	1.23 (0.96–1.58)	0.101	0.76 (0.61–0.95)	0.016	3.14 (2.31–4.27)	<0.001

VAP, ventilator-associated pneumonia; MV, mechanical ventilation; OR, odds ratio; HR, hazard ratio; CI, confidence interval. Ⅰ: adjusted age, gender, ethnicity, coronary heart disease, sepsis (not adjusted in sepsis subgroup), coronary artery disease (CAD), chronic respiratory failure, acute respiratory failure (not adjusted in acute respiratory failure subgroup), chronic obstructive pulmonary disease (COPD) (not adjusted in COPD, subgroup), pneumothorax, emphysema, sequential organ failure assessment (SOFA), charlson comorbidity index (CCI), glasgow coma scale (GCS), systolic blood pressure (SBP), diastolic blood pressure (DBP), heart rate, respiratory rate, temperature, white blood cell (WBC), platelet (PLT), hemoglobin, red blood cell distribution width (RDW), hematocrit, blood urea nitrogen (BUN), creatinine, sodium, bicarbonate, vasopressors, oral care, and opioids. Ⅱ: adjusted age, gender, ethnicity, sepsis (not adjusted in sepsis subgroup), acute respiratory failure (not adjusted in acute respiratory failure subgroup), CAD, COPD (not adjusted in COPD, subgroup), effusion, atelectasis, SOFA, CCI, GCS, SBP, DBP, respiratory rate, temperature, WBC, PLT, RDW, hematocrit, BUN, creatinine, glucose, sodium, chloride, bicarbonate, vasopressors, renal replacement therapy (RRT), oral care, and opioids. Ⅲ: adjusted age, gender, sepsis (not adjusted in sepsis subgroup), CAD, chronic respiratory failure, acute respiratory failure (not adjusted in acute respiratory failure subgroup), COPD (not adjusted in COPD, subgroup), atelectasis, pneumothorax, SOFA, CCI, GCS, heart rate, SBP, respiratory rate, PLT, hematocrit, creatinine, chloride, vasopressors, RRT, oral care, and opioids.

In the Table 6, we excluded all patients without the use of sedative therapy, and explore the effect of different sedative therapy on the prognosis of ICU patients receiving continuous MV across different population. Compared to the use of dexmedetomidine alone, there was no statistically significant relationship between different sedative therapy and the incidence of VAP among patients with COPD, acute respiratory failure and sepsis.

**TABLE 6:** Subgroup analysis about the association of various sedatives administered alone or combination and incidence of VAP, in-hospital mortality, and duration of MV (exclude participants who were not sedated).

Variables	VAP^Ⅰ^		In-hospital mortality^Ⅱ^		Duration of MV^Ⅲ^	
	OR (95% CI)	*P*	HR (95% CI)	*P*	OR (95% CI)	*P*
COPD: no (n = 4,875)						
Dexmedetomidine	Ref		Ref		Ref	
Midazolam	1.20 (0.71–2.03)	0.495	3.01 (1.97–4.59)	<0.001	1.52 (1.11–2.09)	0.010
Propofol	1.69 (1.04–2.75)	0.034	2.70 (1.79–4.08)	<0.001	1.09 (0.81–1.46)	0.583
Combination of midazolam and propofol	1.54 (0.93–2.55)	0.094	2.85 (1.87–4.34)	<0.001	2.24 (1.64–3.06)	<0.001
Combination of midazolam and dexmedetomidine	2.30 (1.10–4.83)	0.027	2.11 (1.17–3.82)	0.013	1.93 (1.09–3.43)	0.025
Combination of propofol and dexmedetomidine	1.74 (1.02–2.96)	0.041	1.71 (1.09–2.70)	0.021	1.63 (1.16–2.29)	0.005
Combination of midazolam, propofol and dexmedetomidine	1.92 (1.13–3.27)	0.017	1.53 (0.97–2.43)	0.069	3.68 (2.55–5.30)	<0.001
COPD: Yes (n = 523)						
Dexmedetomidine	Ref		Ref		Ref	
Midazolam	0.72 (0.14–3.86)	0.706	4.76 (1.32–17.18)	0.017	2.09 (0.83–5.30)	0.119
Propofol	0.91 (0.20–4.04)	0.898	4.43 (1.31–15.03)	0.017	1.73 (0.74–4.02)	0.203
Combination of midazolam and propofol	0.61 (0.13–2.97)	0.540	4.47 (1.27–15.75)	0.020	2.62 (1.05–6.56)	0.039
Combination of midazolam and dexmedetomidine	1.47 (0.13–16.02)	0.753	1.91 (0.29–12.47)	0.497	2.19 (0.49–9.80)	0.305
Combination of propofol and dexmedetomidine	1.06 (0.23–4.76)	0.943	2.24 (0.64–7.81)	0.205	1.63 (0.67–3.98)	0.286
Combination of midazolam, propofol and dexmedetomidine	1.07 (0.22–5.32)	0.930	2.12 (0.56–7.98)	0.267	4.76 (1.70–13.30)	0.003
Acute respiratory failure: no (n = 3,575)						
Dexmedetomidine	Ref		Ref		Ref	
Midazolam	1.28 (0.72–2.27)	0.395	3.59 (2.31–5.58)	<0.001	1.57 (1.12–2.19)	0.008
Propofol	1.63 (0.99–2.67)	0.055	2.97 (1.96–4.52)	<0.001	1.34 (1.00–1.80)	0.051
Combination of midazolam and propofol	1.50 (0.89–2.53)	0.128	2.93 (1.90–4.52)	<0.001	2.24 (1.63–3.09)	<0.001
Combination of midazolam and dexmedetomidine	2.18 (0.94–5.03)	0.069	2.56 (1.37–4.77)	0.003	1.83 (0.95–3.51)	0.069
Combination of propofol and dexmedetomidine	1.83 (1.07–3.12)	0.026	1.88 (1.20–2.96)	0.006	1.80 (1.29–2.51)	<0.001
Combination of midazolam, propofol and dexmedetomidine	2.09 (1.21–3.62)	0.008	1.78 (1.11–2.85)	0.017	4.14 (2.85–6.02)	<0.001
Acute respiratory failure: yes (n = 1823)						
Dexmedetomidine	Ref		Ref		Ref	
Midazolam	0.91 (0.23–3.63)	0.898	1.33 (0.42–4.23)	0.632	1.00 (0.37–2.69)	0.993
Propofol	1.32 (0.34–5.15)	0.687	1.28 (0.40–4.09)	0.677	0.54 (0.20–1.46)	0.226
Combination of midazolam and propofol	1.12 (0.28–4.43)	0.872	1.45 (0.45–4.63)	0.529	1.56 (0.57–4.22)	0.385
Combination of midazolam and dexmedetomidine	2.10 (0.42–10.59)	0.367	0.58 (0.14–2.47)	0.461	1.45 (0.42–5.00)	0.552
Combination of propofol and dexmedetomidine	0.89 (0.19–4.07)	0.881	0.65 (0.17–2.44)	0.523	0.80 (0.26–2.41)	0.687
Combination of midazolam, propofol and dexmedetomidine	1.17 (0.28–4.89)	0.829	0.52 (0.15–1.79)	0.301	2.36 (0.80–6.90)	0.118
Sepsis: no (n = 2006)						
Dexmedetomidine	Ref		Ref		Ref	
Midazolam	2.30 (0.47–11.25)	0.304	3.91 (2.05–7.46)	<0.001	1.41 (0.94–2.11)	0.100
Propofol	2.51 (0.56–11.24)	0.228	3.99 (2.14–7.42)	<0.001	0.92 (0.63–1.35)	0.677
Combination of midazolam and propofol	1.63 (0.33–8.13)	0.553	4.48 (2.36–8.51)	<0.001	1.96 (1.30–2.96)	0.001
Combination of midazolam and dexmedetomidine	2.67 (0.22–32.74)	0.443	2.36 (0.85–6.55)	0.098	2.77 (1.18–6.53)	0.020
Combination of propofol and dexmedetomidine	3.82 (0.78–18.73)	0.099	1.29 (0.60–2.76)	0.514	1.36 (0.86–2.15)	0.193
Combination of midazolam, propofol and dexmedetomidine	4.98 (1.01–24.60)	0.049	0.88 (0.38–2.04)	0.765	2.30 (1.41–3.74)	<0.001
Sepsis: yes (n = 3,392)						
Dexmedetomidine	Ref		Ref		Ref	
Midazolam	1.07 (0.63–1.84)	0.795	2.55 (1.53–4.26)	<0.001	1.82 (1.18–2.81)	0.007
Propofol	1.54 (0.93–2.53)	0.090	2.20 (1.33–3.62)	0.002	1.45 (0.97–2.17)	0.071
Combination of midazolam and propofol	1.40 (0.84–2.34)	0.198	2.30 (1.38–3.81)	0.001	2.80 (1.83–4.27)	<0.001
Combination of midazolam and dexmedetomidine	2.19 (1.04–4.61)	0.039	1.91 (0.96–3.81)	0.065	1.77 (0.89–3.49)	0.101
Combination of propofol and dexmedetomidine	1.52 (0.89–2.60)	0.126	1.75 (1.03–2.97)	0.039	1.98 (1.27–3.09)	0.003
Combination of midazolam, propofol and dexmedetomidine	1.67 (0.97–2.86)	0.064	1.66 (0.97–2.85)	0.065	5.78 (3.53–9.46)	<0.001

VAP, ventilator-associated pneumonia; MV, mechanical ventilation; OR, odds ratio; HR, hazard ratio; CI, confidence interval. Ⅰ: adjusted age, gender, ethnicity, coronary heart disease, sepsis (not adjusted in sepsis subgroup), coronary artery disease (CAD), chronic respiratory failure, acute respiratory failure (not adjusted in acute respiratory failure subgroup), chronic obstructive pulmonary disease (COPD) (not adjusted in COPD, subgroup), pneumothorax, emphysema, sequential organ failure assessment (SOFA), charlson comorbidity index (CCI), glasgow coma scale (GCS), systolic blood pressure (SBP), diastolic blood pressure (DBP), heart rate, respiratory rate, temperature, white blood cell (WBC), platelet (PLT), hemoglobin, red blood cell distribution width (RDW), hematocrit, blood urea nitrogen (BUN), creatinine, sodium, bicarbonate, vasopressors, oral care, and opioids. Ⅱ: adjusted age, gender, ethnicity, sepsis (not adjusted in sepsis subgroup), acute respiratory failure (not adjusted in acute respiratory failure subgroup), CAD, COPD (not adjusted in COPD, subgroup), effusion, atelectasis, SOFA, CCI, GCS, SBP, DBP, respiratory rate, temperature, WBC, PLT, RDW, hematocrit, BUN, creatinine, glucose, sodium, chloride, bicarbonate, vasopressors, renal replacement therapy (RRT), oral care, and opioids. Ⅲ: adjusted age, gender, sepsis (not adjusted in sepsis subgroup), CAD, chronic respiratory failure, acute respiratory failure (not adjusted in acute respiratory failure subgroup), COPD (not adjusted in COPD, subgroup), atelectasis, pneumothorax, SOFA, CCI, GCS, heart rate, SBP, respiratory rate, PLT, hematocrit, creatinine, chloride, vasopressors, RRT, oral care, and opioids.

## Discussion

In this study, we evaluated the correlation between sedative therapy and the incidence of VAP, in-hospital mortality, and duration of MV among patients receiving MV. Among a total of 11,491 patients, the overall incidence rate of VAP was 9.87%, while the overall in-hospital mortality stood at 21.11%. Additionally, the median duration of MV was recorded as 70.50 h with a quartile range between 57.00 and 99.00 h. The findings of our study indicated that the administration of sedatives, in comparison to non-administration, may potentially elevate the risk of VAP, decrease in-hospital mortality (except for propofol alone), and prolong the duration of MV. However, when compared to the administration of dexmedetomidine alone, the utilization of midazolam or propofol as standalone sedatives or in combination for patients on mechanical ventilation may potentially lead to an increased incidence of VAP, higher in-hospital mortality, and prolonged duration of MV, suggesting that dexmedetomidine alone may be beneficial for outcomes of patients receiving MV.

In line with prior research, the administration of sedatives generally impacts the incidence of VAP in ICU patients undergoing MV (Dou et al., 2020). Our study findings indicated that administration of propofol or dexmedetomidine alone increased the risk of VAP compared to patients not receiving these medications. Similar results were observed in combination of midazolam and dexmedetomidine, combination of propofol and dexmedetomidine, combination of midazolam, propofol and dexmedetomidine. Besides, the utilization of certain sedatives has been observed to potentially mitigate in-hospital mortality compared to patients not receiving these medications. However, the utilization of sedatives exhibited a positive correlation with the duration of MV. For ICU patients receiving MV, the administration of sedatives is essential for ensuring patient comfort and safety during treatment (Panahi et al., 2018; Jabaudon et al., 2022).

As commonly used sedatives, midazolam, propofol, and dexmedetomidine have been investigated for their effects on ICU patients (Jakob et al., 2012). A previous systematic review and meta-analysis suggested that dexmedetomidine was associated with a reduction in the duration of MV among mechanically ventilated patients with sepsis (Liu et al., 2022). Similarly, sedation with dexmedetomidine significantly decreased the duration of MV and lowered the risk of delirium in cardiac surgery patients compared to propofol (Heybati et al., 2022). A multicenter and double-blind trial also found that the clinical outcomes of adult patients with mechanically ventilated sepsis did not differ between those receiving dexmedetomidine and those receiving propofol (Hughes et al., 2021). In the study conducted by Tekeli et al., it was demonstrated that the combination of dexmedetomidine and propofol exhibited superior efficacy in the upper gastrointestinal system endoscopy (Tekeli et al., 2020). The sedation protocol for mechanically ventilated patients in the ICU still lacks sufficient research, thus, a comparative analysis of individual sedatives or combination is needed to determine which ones can enhance outcomes in mechanically ventilated patients in the ICU. For this current study, we investigated the correlation between midazolam alone, and propofol alone, as well as the relationship between the use of both and the combination of all three and outcomes of mechanically ventilated patients in the ICU. The findings also revealed that, taking use of dexmedetomidine alone as reference, the use of midazolam and propofol alone or in combination may increase the incidence of VAP, in-hospital mortality, and duration of MV. Overall, dexmedetomidine alone may present as a favorable prognostic option for patients. Our results may be helpful for the rational use of sedative in critically ill patients with mechanical ventilation. However, the results need to be verified in the future by multicenter randomized controlled trials.

The limitations of the study should be underscored. Firstly, as a single-center retrospective study, caution should be exercised in interpreting the results due to potential selection bias. Secondly, given the nature of observational studies, our findings do not establish a causal relationship of sedation and prognosis of ICU patients receiving continuous MV. Furthermore, this study was limited by the MIMIC database in terms of recording, and data on the dosage and duration of sedation drugs could not be obtained, necessitating further verification through a prospective, multi-center study.

## Conclusion

Compared to the use of midazolam and propofol alone or in combination, administering dexmedetomidine alone as a sedative therapy for mechanically ventilated patients may potentially decrease the incidence of VAP and in-hospital mortality, while also resulting in a shorter duration of mechanical ventilation. Further research is warranted to explore the potential application of dexmedetomidine alone in mechanically ventilated patients within ICU.

## Data Availability

Publicly available datasets were analyzed in this study. This data can be found here: MIMIC-IV database, https://mimic.physionet.org/iv/.
