# Supplementary figures and images for: Effect of different sedatives on the prognosis of patients with mechanical ventilation: a retrospective cohort study based on MIMIC-IV database
Source: Front Pharmacol. 2024 Jul 18;15:1301451. doi: 10.3389/fphar.2024.1301451 (PMC11291308; doi:10.3389/fphar.2024.1301451)

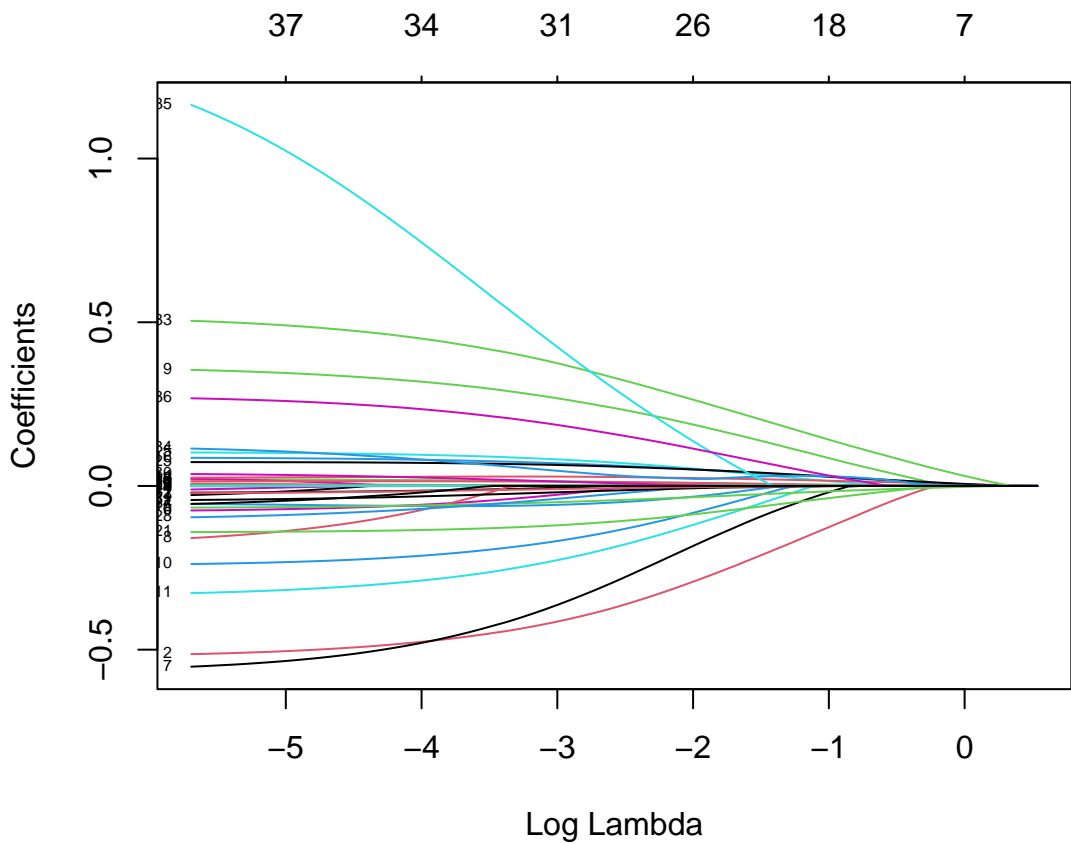

Supplement: Supplementary file 1 [file DataSheet2.PDF]

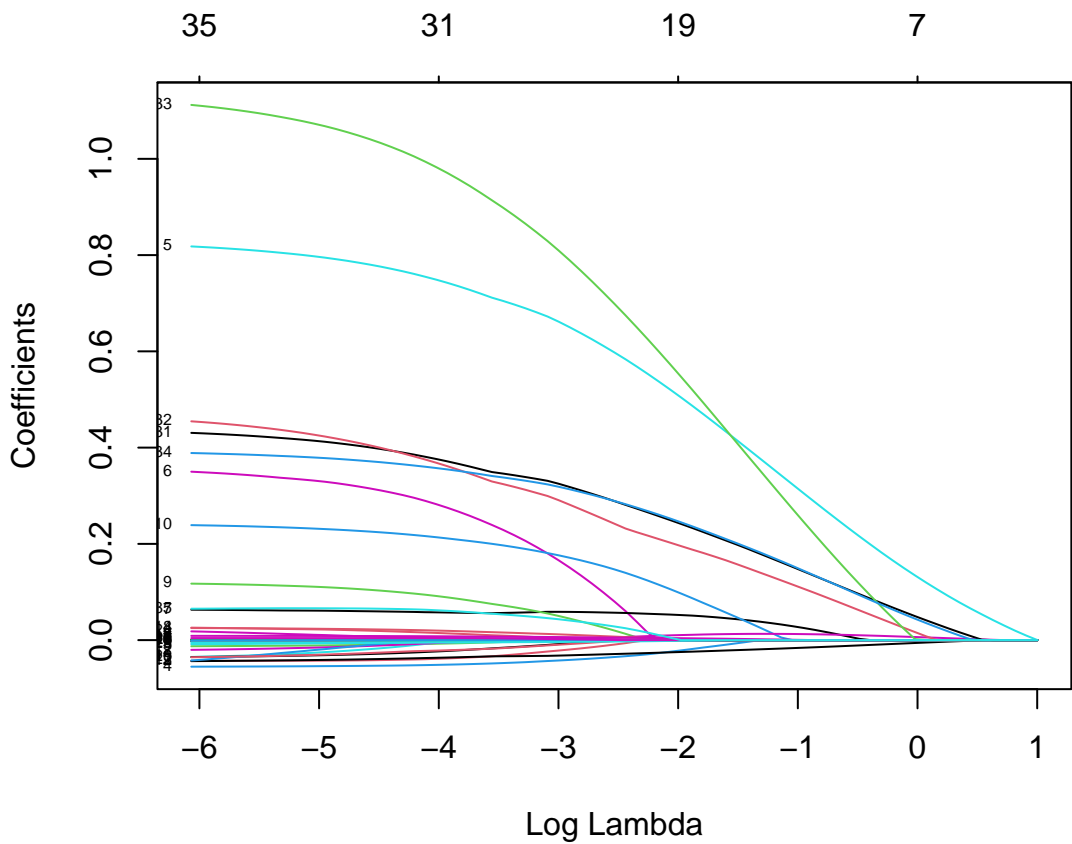

Supplement: Supplementary file 3 [file DataSheet3.PDF]

Coefficients

33

31

22

3

2.0

1.5

1.0

0.5

0.0

-6

-4

-2

0

Log Lambda

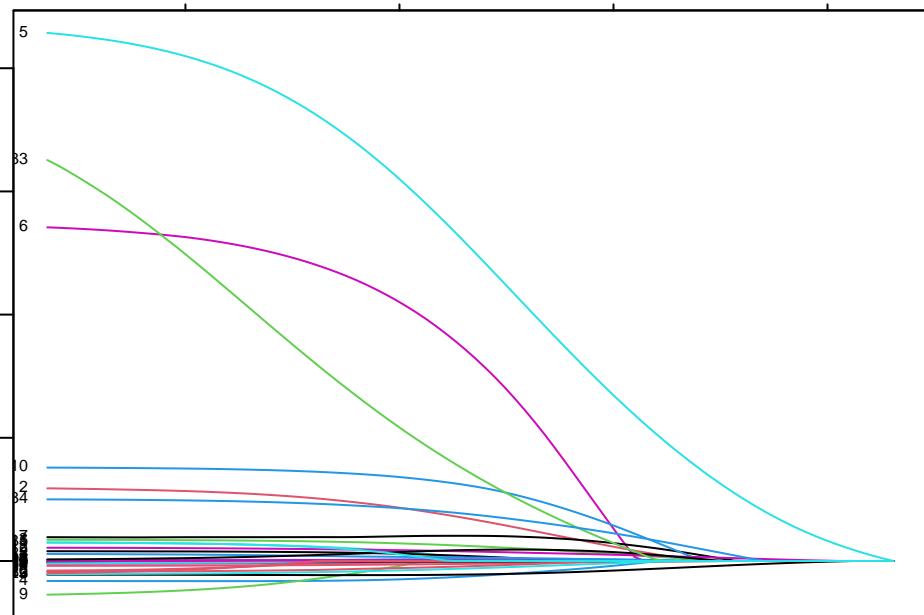

Supplement: Supplementary file 4 [file DataSheet1.PDF]
