# Supplementary material for: Effect of different sedatives on the prognosis of patients with mechanical ventilation: a retrospective cohort study based on MIMIC-IV database
Source: Front Pharmacol. 2024 Jul 18;15:1301451. doi: 10.3389/fphar.2024.1301451 (PMC11291308; doi:10.3389/fphar.2024.1301451)
Supplement: Supplementary file 2 [file Table1.DOCX]

**Supplemental Table 1 The difference analysis for before and after data interpolation**

| **Variables** | **After interpolation (n=11491)** | **Before interpolation (n=11491)** | ***P*** |
| --- | --- | --- | --- |
| SBP, mmHg, Mean ± SD | 119.13 ± 24.52 | 119.13 ± 24.52 | 0.997 |
| DBP, mmHg, Mean ± SD | 65.06 ± 17.17 | 65.06 ± 17.17 | 0.990 |
| Respiratory rate, times/min, Mean ± SD | 20.65 ± 6.05 | 20.65 ± 6.05 | 0.986 |
| Temperature, ℃, Mean ± SD | 36.72 ± 0.95 | 36.72 ± 0.95 | 0.864 |
| WBC, K/uL, M (Q1, Q3) | 11.90 (8.50, 16.60) | 11.90 (8.50, 16.60) | 0.986 |
| PLT, K/uL, M (Q1, Q3) | 187.00 (131.00, 257.00) | 187.00 (131.00, 257.00) | 0.878 |
| Hemoglobin, g/dL, Mean ± SD | 10.52 ± 2.18 | 10.53 ± 2.18 | 0.963 |
| RDW, %, Mean ± SD | 15.38 ± 2.39 | 15.37 ± 2.39 | 0.930 |
| Hematocrit, %, Mean ± SD | 32.10 ± 6.50 | 32.10 ± 6.50 | 1.000 |
| BUN, mg/dL, M (Q1, Q3) | 21.00 (14.00, 36.00) | 21.00 (14.00, 36.00) | 0.970 |
| Creatinine, mg/dL, M (Q1, Q3) | 1.10 (0.70, 1.70) | 1.10 (0.70, 1.70) | 0.992 |
| Glucose, mg/dL, M (Q1, Q3) | 135.00 (110.00, 173.00) | 135.00 (110.00, 173.00) | 0.903 |
| Sodium, mEq/L, Mean ± SD | 138.26 ± 5.33 | 138.27 ± 5.34 | 0.967 |
| Chloride, mEq/L, Mean ± SD | 103.91 ± 6.76 | 103.91 ± 6.75 | 0.992 |
| Bicarbonate, mEq/L, Mean ± SD | 22.94 ± 5.06 | 22.94 ± 5.06 | 0.975 |

SBP, systolic blood pressure; DBP, diastolic blood pressure; WBC, white blood cell; PLT, platelet; RDW, red blood cell distribution width; BUN, blood urea nitrogen.
